# Cell tracking and therapy evaluation of bone marrow monocytes and stromal cells using SPECT and CMR in a canine model of myocardial infarction

**DOI:** 10.1186/1532-429X-11-11

**Published:** 2009-04-27

**Authors:** Gerald Wisenberg, Katie Lekx, Pam Zabel, Huafu Kong, Rupinder Mann, Peter R Zeman, Sudip Datta, Caroline N Culshaw, Peter Merrifield, Yves Bureau, Glenn Wells, Jane Sykes, Frank S Prato

**Affiliations:** 1 Department of Medicine, University of Western Ontario, Ontario, Canada; 2 Department of Medical Biophysics, University of Western Ontario, Ontario, Canada; 3 Department of Anatomy and Cell Biology, University of Western Ontario, Ontario, Canada; 4 Department of Medicine, University of Ottawa, Ontario, Canada

## Abstract

**Background:**

The clinical application of stem cell therapy for myocardial infarction will require the development of methods to monitor treatment and pre-clinical assessment in a large animal model, to determine its effectiveness and the optimum cell population, route of delivery, timing, and flow milieu.

**Objectives:**

To establish a model for a) in vivo tracking to monitor cell engraftment after autologous transplantation and b) concurrent measurement of infarct evolution and remodeling.

**Methods:**

We evaluated 22 dogs (8 sham controls, 7 treated with autologous bone marrow monocytes, and 7 with stromal cells) using both imaging of ^111^Indium-tropolone labeled cells and late gadolinium enhancement CMR for up to12 weeks after a 3 hour coronary occlusion. Hearts were also examined using immunohistochemistry for capillary density and presence of PKH26 labeled cells.

**Results:**

In vivo Indium imaging demonstrated an effective biological clearance half-life from the injection site of ~5 days. CMR demonstrated a pattern of progressive infarct shrinkage over 12 weeks, ranging from 67–88% of baseline values with monocytes producing a significant treatment effect. Relative infarct shrinkage was similar through to 6 weeks in all groups, following which the treatment effect was manifest. There was a trend towards an increase in capillary density with cell treatment.

**Conclusion:**

This multi-modality approach will allow determination of the success and persistence of engraftment, and a correlation of this with infarct size shrinkage, regional function, and left ventricular remodeling. There were overall no major treatment effects with this particular model of transplantation immediately post-infarct.

## Background

Beginning in 2001, tremendous excitement was stimulated regarding the potential to "heal" or reduce the extent of necrosis following myocardial infarction, using transplanted progenitor cells. These early small animal studies demonstrated a remarkable degree of reduction of myocardial injury and improvement in left ventricular function [1-8]. Such enthusiasm was generated that a number of clinical trials were conducted [9-14]. However, the inconsistent and limited treatment effects in these recent trials have tempered this enthusiasm [15,16].

Therefore, the question persists as to whether the early results can be translated into the clinical realm. More recent animal studies have cast further doubt regarding the degree of engraftment, whether bone-marrow-derived cells differentiate into cardiomyoctes [17,18], and whether any therapeutic effect occurs. Assuming benefit, there are several unanswered questions re: specific cell lines, optimum route of delivery, timing, and regional flow environment.

Resolution of these will require pre-clinical evaluation in a large animal model to monitor the degree of engraftment, and correlation with measurable treatment effects on infarct evolution, including left ventricular remodeling.

There are potentially a number of different approaches for in vivo cell tracking: paramagnetic iron oxide particle labeling imaged with cardiovascular magnetic resonance (CMR) [19-25]; radiolabeling of reporter probes [26-29]; and incorporation of radioactively labeled compounds into transplanted cells with in vivo PET or SPECT [30]. In our own hands, the use of a reporter probe in a large animal model (dog), did not appear to be feasible because of high non-specific background uptake [31].

Cell labeling techniques are commonly applied to hematopoetic cells using technetium, indium-based compounds or fluorinated-2-de-oxy-glucose [32-36]. Indium labeling has become established for tracking marrow-derived cells in vivo [36,37], and we have chosen this method to establish the presence, and degree of retention of cells. A recent in vitro and phantom study in our laboratory indicated that as few as 3,600 cells may be detected with 111In SPECT [38]. This sensitivity is dependent on a maximum average concentration of radioactivity of 111In of 0.14 Bq/cell which we have shown can be safely incorporated without affecting viability, function, or proliferative capacity [38]. However, another laboratory has suggested that much higher radioactive loading is possible [39].

This study was undertaken to establish a method to concurrently use SPECT and CMR to 1) monitor cell engraftment, and 2) the effects of transplantation on infarct size, regional function, and remodeling indices, in a canine model of reperfused anterior myocardial infarction using bone marrow-derived monocytes (BMMC's) [40-43] or stromal (mesenchymal) cells [44-47], which have been reported to have favorable effects on myocardial regeneration. The goals of this study were primarily to demonstrate the ability to perform these assessments in the same animal, and to determine the evolution of infarct-related changes. By restricting the development and application of techniques and technologies in a large animal model to those already approved for human use, translation to human use is assured.

## Methods

### Animal Preparation

Adult female bred-for-research hounds were used. All procedures were approved by the Animal Care Committee of the University of Western Ontario, and were performed according to the Guide of the Care and Use of Experimental Animals of the Canadian Council on Animal Care and Use of Laboratory Animals, National Research Council. We used a 3 hour left anterior descending occlusion/reperfusion model with cells injected 3 hours after reperfusion, i.e. 6 hours after the onset of coronary occlusion. The animals subsequently underwent serial imaging for 12 weeks, and then were sacrificed.

### Cell Harvesting and Labeling

#### Preparation of Bone Marrow Mononuclear Cells and Bone Marrow Stromal Cells

In anticipation of autologous transplantation, under general anesthesia, bone marrow was aspirated from either the sternum or humerus with a heparinized syringe. The marrow aspirate was diluted 1:3 with PBS and 8 mls was layered over a 4 ml Ficoll cushion and centrifuged for 20 minutes at 430 g to pellet RBCs and platelets. BMMCs were collected from the Ficoll/serum interface, pelleted at 430 g for 8 minutes and the pellet (containing RBCs and BMMCs) resuspended in 10 mls PBS. Three volumes of lysis buffer (high osmolarity ammonium chloride) were added to the mixture and incubated on ice for 7 minutes to selectively lyse RBCs, then centrifuged at 430 g for 8 minutes and the white BMMC pellet resuspended in 2 mls PBS containing 5% FBS. Cells were counted on a hemacytometer, washed with PBS and either used directly for radioactive labeling and injection on the day of isolation (BMMC) or cultured on plastic tissue culture dishes after further isolation (stromal) (Falcon, VWR, Mississauga, ON) in growth medium consisting of DMEM, 10% FBS, glutamate and penicillin/streptomycin.

To obtain sufficient stromal cells for transplantation, these cells were culture expanded for approximately 14 days. Specifically, the growth medium originally containing the BMMC's was changed twice weekly and the non-adherent cells discarded. With washing, the hematopoietic cells were washed away, and only the remaining adherent stromal cells were retained. No unique membrane marker was used for identifying stromal cells, but they are generally considered to lack the c-kit, CD34 and CD45 markers characteristic of Hematopoietic Stem Cells (HSC) [48,49]. The stromal cell population is highly heterogeneous with respect to biomarkers and may contain anywhere from 0.01 to 0.001% mesenchymal stem cells (MSCs) [48]. In future experiments, these cells may be enriched by FACS using MSC-specific markers such as CD13, CD29 and CD44 [49]

#### 111In Tropolone Labeling of Bone Marrow Cells

We previously have described 111In tropolone labeling of cells [38]. Briefly, cells were incubated with 111In-tropolone in phosphate buffered saline (PBS) for 30 minutes at 37°C. Then, cells were centrifuged at 430 g for 10 min at 20°C. The supernatant was discarded and the pellet was washed three times with PBS as described above. Typical labeling efficiencies were ~60%. The combination of labeling efficiency, number of cells incubated and dose of radioactivity ensured that cells were labeled with < 0.14 Bq/cell, the dose we have previously demonstrated to cause no adverse effects on cell viability and proliferation [38] Labeled cells were typically transplanted by direct injection within 90 minutes of the start of labeling.

We have investigated the correspondence between the 111In signal detected at the transplantation site and the contribution to that signal by a) ^111^In inside viable cells, b) ^111^In released by dead cells which have not been cleared, and c) ^111^In leaked from viable cells and not cleared [50]. We have discovered that there is a consistent initial clearance of 111In with a biological half life of ~2 hours attributable to viable cells rapidly leaving the injection site. This initial clearance is followed by a slower clearance attributed to the biological half life provided the true biological half life of the transplanted cells is >1 and <20 days. The lower limit is set by the rate of clearance of 111In labeled cellular debris and the upper limit by the rate at which 111In leaks from viable transplanted cells. The experiments performed in our laboratory and reported by Blackwood et al [50] indicate that Indium released by either viable or non-viable cells is not taken up to any degree either by these stem cells or a rat embryonic cardiomyoblast H9c2 cell line [50], and is rapidly cleared from the site of injection.

#### Labelling BMMC and Stromal Cells with PKH26

PKH26 is a lipophilic marker inserted into the membranes of viable cells [51], which cannot be passed from cell to cell, and effectively labels the cell membrane. This marker provided a means of identifying the transplanted cells histologically following sacrifice. BMMC's and stromal cells were completely trypsinized with a 1:50 dilution of 20 mg % trypsin (Gibco/BRL, Burlington, Ontario, Canada) for 10 min. Cells were washed once by centrifugation for 8 min at 800 g followed by resuspension in complete media with serum. This wash was then repeated using Dulbecco's MEM (Gibco/BRL, Burlington, Ontario, Canada) without serum. After cells were centrifuged a third time, they were resuspended in 1 ml of Diluent C (Sigma Chemical Co, St Louis, Missouri, USA) according to the manufacturer's instructions. The PKH26 membrane label (Sigma Chemical Co, St. Louis, Missouri, USA) was prepared to a concentration of 15 ul of PKH26 stock (in ethanol) in 1 ml of diluent C, and then added to the cell suspension. Cells were incubated at room temperature (RT) for 4 min with the tube inverted every minute. Following incubation, an equal amount of horse serum (HyClone Labs Inc, Logan, Utah, USA) was added and cells incubated for one minute. An equal volume of complete media was added and cells were centrifuged as usual. Cells were then washed two times with complete media to remove any unbound label.

### Surgical Preparation

Dogs were anesthetized using intravenous Propofol (1 ml/kg), intubated and ventilated with oxygen enriched room air, and maintained with Isofluorane (2%). Following thoracotomy, the left anterior descending coronary artery was identified and ligated for 3 hours using a snare, and then released. Eight control animals received only injections of normal saline into the central and peri-infarct areas 3 hours after release of the snare (6 hours from the onset of the occlusion). Seven animals received an injection of 2–3 × 10^7 ^BMMC's, and 7 animals, 1.5–1.7 × 10^7 ^stromal cells, also 3 hours after snare release. These animals were then imaged on a regular basis (as described below) for 12 weeks and then sacrificed with potassium chloride.

An additional five animals were studied to establish the retention of the PKH26 cell labeling (3 animals with BMMC's) and parameters for SPECT (2 animals). The 3 PKH26 animals were sacrificed at three weeks. For SPECT, the animals were injected with stromal cells and imaged at day 0 (surgery), 4, 7, 10 and 14 days.

### Cell Transplantation

#### BMMC experiments

On the day of surgery, marrow was harvested and the cells were separated and co-labeled with PKH-26 and ^111^Indium-tropolone. Cells were also mixed in India ink for both gross and microscopic determination of the sites of injection. We did not demonstrate any harmful effects related to India ink. A small aliquot of cells was not injected but maintained in culture and monitored daily for 2 weeks. Autologous cells were injected directly into the infarct and peri-infarct region (by visual assessment of both discoloration and regional wall motion at the epicardial surface) at multiple sites (8–10) using a 25-gauge needle.

#### Stromal cell transplantation

After the cells had been culture expanded for two weeks, they were injected directly into the infarct and peri-infarct regions, and imaging began. As was the case for the BMMC's, a small aliquot of cells was kept in culture and monitored.

### Imaging Protocols and Analysis

#### CMR

CMR was performed on the day of surgery, and then weekly to 8 weeks, and then at 10 and 12 weeks. CMR was performed on a Siemens Avanto 1.5 T clinical scanner using a rigid radiofrequency transmit/receive coil (Siemens, CP Head coil). A mid-ventricular, transaxial gradient-echo 'scout' image was used to locate the long-axis and short-axis (SAX) image-planes. Cine CMR for the assessment of wall motion was obtained using a segmented cineFLASH sequence with 5 lines per segment, 8–12 segments per beat, TR/TE 10/4.8 ms, α = 20°, slice thickness 8 mm, and a rectangular field of view (FOV, 175–250 × 400 mm).

For the assessment of infarct size, each imaging session used a 0.2 mmol/kg bolus of Gd-DTPA (Magnevist, Berlex Canada, Lachine, Québec, Canada), followed by a constant infusion of 0.004 mmol/min/kg for 45–60 min, to ensure a steady state [52-54]. This method has been validated in our laboratory in both canine and clinical settings to provide excellent delineation of the extent of scar and correlation with histological measurement of infarct size [53]. Using this method removes the dependence on the timing of imaging as a variable affecting the increase in signal within the infarct zone as equilibrium is established between blood and tissue concentrations of Gd-DTPA [53]. The imaging sequence used for infarct size evaluation was a segmented inversion-recovery turboFLASH (irTFL, TR/TE 8.0/4.0 ms, α = 25°, TI chosen iteratively to null the normal myocardium), acquired after at least 30 min continuous infusion, synchronized to the cardiac cycle (at end diastole) and with breath holding (respirator turned off). A stack of 6–7 (8 mm thick) contiguous short-axis irTFL and cine MR images was acquired, in order to obtain full LV coverage.

#### Left-ventricular Wall Motion Analysis

Cine CMR images were used to qualitatively assess left-ventricular wall motion for every slice position and time-point in each animal using a validated method [55]. To briefly review, each short-axis slice was divided into six segments (septal, infer-septal, antero-septal, lateral, antero-lateral, and infero-lateral). For each segment of every slice, a subjective quantitative score assesing wall motion was assigned. Hyperkinetic wall motion was assigned a score of 7, normal, 6, mildly hypokinetic 5, moderately hypokinetic 4, severely hypokinetic 3, akinetic 2, and dyskinetic 1. Each individual cine was interpreted by one of three experienced cardiologists (GW, PZ, and SD), blinded to treatment and time-point of each study. Only those segments with a baseline (immediately post-infarction) wall motion score of 4 (moderately hypokinetic) or less (more severe) were analyzed for subsequent treatment effects. Also, as there was considerable variation in the extent of wall motion abnormalities initially (the number of segments affected), only the average score for each individual animal at any given time point were used for treatment comparisons.

#### Analysis of Infarct Size

We have previously reported in detail our method for the determination of infarct size [53,56] which is modeled after the initial work of Kim [57]. For each irTFL image, the endo- and epi-cardial borders were traced manually using Analyze AVW software [58] (Mayo Clinic, Rochester, Maine, USA). In a remote region, the signal intensity (SI) was sampled and used to apply a semi-automatic segmentation of the LV: a region was deemed 'infarcted' if it consisted of pixels with SI >2 SD above that of remote (i.e. normal) myocardium [57]. The total number of infarcted pixels for all slices was determined and expressed as a percentage of the total in the LV. The latter parameter, corrected for absolute volumes, was used for total left ventricular volume (mass) and the endocardial contour allowed determination of the end-diastolic volume.

#### Nuclear Medicine Imaging and Analysis

In the two animals imaged five times in 14 days, the purpose was to determine a) the period of time over which ^111^In could be detected, and b) the anatomic location of the cells with respect to the infarct during that time interval. In one animal, stromal cells were injected into the infarct while in the second animal, cells were injected into both the infarct as well as the normal tissue at a distance of 3 cm from the first injection site. For the second animal, the signals from the two injection sites were analyzed separately.

Imaging was performed on a dual-head MillenniumMG gamma camera (General Electric Healthcare Technologies, Waukishaw, WI) using medium-energy general purpose collimators. Images were acquired on surgery day (day 0), and repeated on days 4, 7, 10 and 14. Initially, a 20-min whole-body scan was acquired to assess the extent of radio-tracer distribution. An ^111^In SPECT image, centered on the heart, was then acquired with a scan time starting at 40 minutes on day 0 and gradually increasing to 4 hours on day 14. Immediately following the ^111^In imaging, 783 ± 70 MBq of ^99m^Tc-labeled sestamibi was injected. A 30-min ^99m^Tc SPECT image was acquired one hour after injection to both assess myocardial perfusion and provide an anatomical context for the ^111^In images.

After background correction, the ^111^In projection data were reconstructed incorporating resolution compensation, and then filtered with a 5.4 mm FWHM Gaussian filter. The reconstructed image array was 128 × 128 × 128 (2.7 mm isotropic voxel size). The sestamibi images were reconstructed in a similar fashion and co-registered using the Analyze AVW software package [58](Mayo Clinic, Rochester, MN). A volume of interest (VOI) was defined on the weighted sum of the five ^111^In images by thresholding the indium volume at 3% of the maximum value. The number of counts in this volume at each of the five imaging days was used to determine the in vivo time-activity curve (TAC) of the ^111^In activity. Additionally the TAC of the ^111^In signal from the single-site animal was calculated directly from the projection data (total counts minus the background) and used to confirm the value obtained from the images reconstructed from the projection data, supporting our image-based approach.

The results from these preliminary dog experiments gave consistent results. For all three injection sites, the signal decayed mono-exponentially with similar biological half-lives of approximately 5 days: (5.8, 5.1, and 5.6 days respectively). Thus, we were able to simplify subsequent acquisition and analysis. To determine the biological half life of the cells at the injection site, we performed whole body ^111^In scanning at only 3 time points: within 30 minutes of cell injection, 7 days later and again 14 days after the transplantation. ^111^In scanning was done in three of the seven dogs given BMMC's and four of the seven dogs given stromal cells. The logistics of doing both ^111^In-scanning and CMR during the same anesthetic period limited the number of animals imaged with ^111^In, although all had CMR as previously described.

The following analysis was done on the whole body scans. On surgery day, the counts for the whole body and region over the heart were calculated and background corrected. The ratio of the activity inside the heart over the total activity measured in the body was taken as the percentage of ^111^In (stem cells) that remained in the heart after injection. On day 7 and 14, the counts over the region of the heart were calculated and background and decay corrected. The ratio of the decay-corrected counts in the heart over the total activity measured on surgery day was taken as the percent of In-111 (stem cells) that remained within the heart.

### Histological Analysis

#### Detection of fluorescently labeled cells at injection sites

Immediately following sacrifice, hearts were removed and cut transversely from apex to base into 4–5 rings. Injection sites were identified by India Ink staining and 1 cm blocks of tissue containing injection sites were dissected and snap frozen in OCT by immersion in melting isopentane at -80°C. Blocks were then cut at 10 μM using a Leitz cryostat and representative sections (those adjacent to the India ink marker) were stained with hematoxylin and eosin. Serial sections were analysed for PKH26 fluorescence using a Zeisss Axiophot fluorescence microscope at 10×, 40×, and 630× magnification using the TRITC filter series to detect red fluorescence.

In the 3 preliminary studies, not included in data analysis of treatment effect, dogs were euthanized at 3 weeks post-injection (rather than 12 weeks) and injection sites analyzed for PHK26 labeled cells using a Leitz Axioplan fluorescence microscope with the TRITC filter series. Serial sections were analysed for Myosin Heavy Chain (MyHC) immunofluorescence using a cardiac MyHC specific monoclonal antibody (Mab 4A9). Hoesch 33258 was used to stain nuclei.

#### Blood vessel density in heart sections

In light of numerous reports that stem cell injections can promote angiogenesis through a paracrine mechanism [59-61], injection sites from 6 control, 5 BMMC and 7 stromal injected dogs were examined for capillary density using alkaline phosphatase histochemistry to identify endothelial cells. Capillaries were identified as dark purple structures in phase contrast microscopy, either as spots (in cross section) or short tubes (in longitudinal section). Capillary density was quantified, as previously described by Oshima et al [62]. Two injection sites were analysed for each animal and 5 fields were counted at 400× magnification for each injection site. Fields were typically located in the myocardium near the infarct border, and were randomly chosen using a random number generator (ie a phone book). Vessel density was calculated per mm^2^.

### Statistical Analysis

For each time point, an analysis of variance corrected for repeated measures was conducted to determine significant group differences. When a significant result was observed, Tukey tests determined which groups were significantly different. Results were considered significant when the probability of a type one error was less than 0.05. All data are presented as means plus/minus standard error of the means (SEM).

In order to determine if the evolving changes in the extent of scar was dependent on the initial infarct size at surgery, a Pearson Product Moment Correlation was conducted for the initial extent of the infarct at surgery vs. the percent change in infarct size from surgery at all time points. A significant correlation would suggest that normalization would not be appropriate.

## Results

### Cell Viability

We have previously shown that bone marrow cells incubated with 0.9 MBq of ^111^In or less per 5 million cells had 100% viability over 14 days in culture (0.14 Bq/cell with a labeling efficiency of 80%) [38]. We also demonstrated an excellent correlation (*r *= 0.99, *P *< 0.01) between the subsequent proliferation rate of cells labeled with 0.9 MBq ^111^In-tropolone and that of unlabelled control cells. In the present study,**t**he aliquots of BMMCs and stromal cells kept in vitro showed normal proliferation and viability 2 weeks after they were labeled with ^111^Indium-troplone and PKH-26, and mixed with India ink (results not shown).

### In Vivo Indium Imaging Data

**Figure 1:**
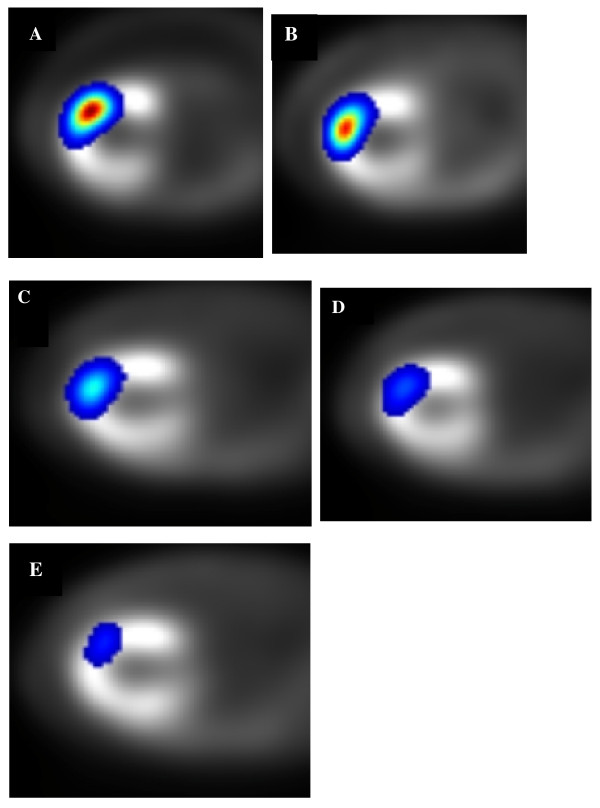
**Serial Transaxial SPECT images following Indium labeling and intravenous Tc-99m MIBI**. Panels A-E respectively are fused images of Tc-MIBI and 111In-labeled stromal cells in a dog at day 0, 4, 7, 10 and 14.

Fig. 1A–E shows the location of the ^111^In radioactivity at the injection site co-registered with the perfusion deficit on the 99mTc MIBI images in the dog imaged at several time points through to day 14.

The biological clearance of cells from the injection site was described by a mono-exponential function giving the following results: for the BMMC injected dogs 5.7 days, 4.4 days and 4.4 days giving an average of 4.8 days; for the four stromal cell injected dogs: 4.6 days, 6.1 days, 5.9 days and 4.7 days giving an average of 5.3 days.

### MR Assessment of Scar Shrinkage, Wall Motion, Ventricular Mass and End Diastolic Volume (Figs 2, 3, 4 and 5)

**Figure 2:**
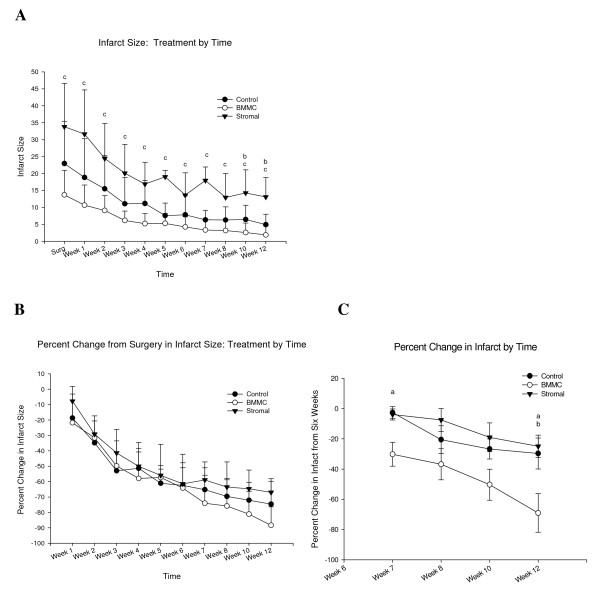
**Absolute and relative changes in infarct size over time**. There was a progressive decline in both absolute (A) and relative (B) CMR measured infarct size, in comparison to baseline, for controls, and both treatment groups. Values are means ± SE. One way analyses of variance showed that significant group differences were observed in relative infarct size changes. Posthoc Tukey Tests showed significant paired group differences at 12 weeks when the 6 week time point was used as the reference point for further change. a-Control vs. BMMC p = 0.046, b-Stromal vs. BMMC p = 0.032,

**Figure 3:**
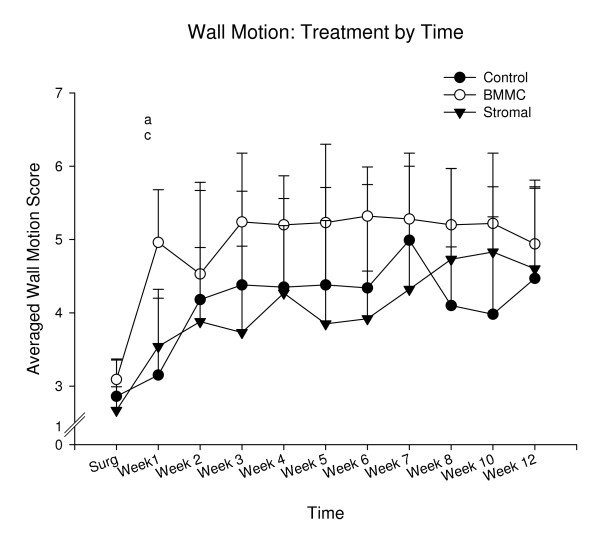
**Changes in regional wall motion scores**. Although there was a progressive improvement in regional function in the infarct and peri-infarct areas by almost 2 wall motion scores, there was no difference between treatments *at 12 weeks*. Separate one way analyses of variance showed that significant group differences were observed at week one only, F(2,16) = 8.08, p < .01. Posthoc Tukey Tests showed significant paired group differences between a = Controls and BMMC, and c = Stromal and BMMC.

**Figure 4:**
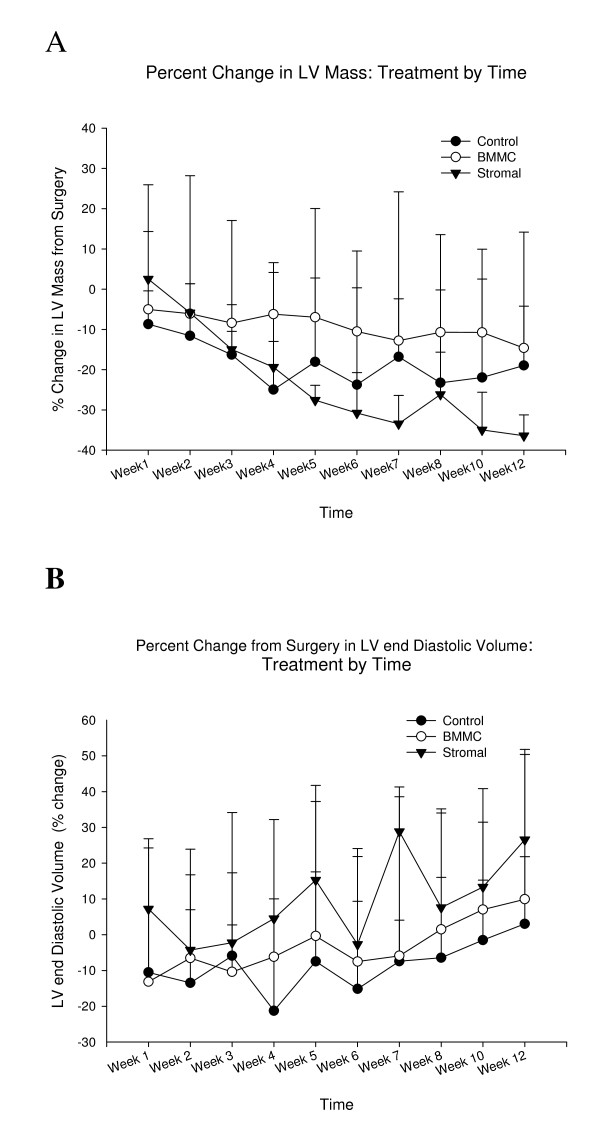
**Changes in left ventricular volume and enddiastolic volumes**. A The stromal cell animals had a significant decline in left ventricular volume (total mass) in comparison to both controls, and BMMC from 8 weeks through 12 weeks: b- stromal different from controls c- stromal different from BMMC. There were small increases in endiastolic volume over time but there were no differences between treatments. The first data point on the graph is at the first week following surgery relative to the volume on the day of surgery.

**Figure 5:**
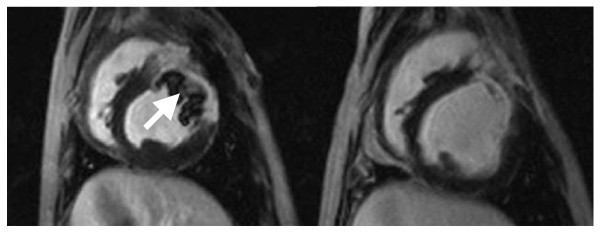
**Late gadolinium enhanced end diastolic images from one of the stromal cell animals on the day of infarction, left, and at 12 weeks, right**. The initial image demonstrates a large infarct with a central area of no enhancement. Such a pattern is often seen with extensive microvascular injury leading to "no reflow" and failure of delivery of tracer to the central zone of infarction. The 12 week image demonstrates a considerable reduction in the extent of infarction, and thinning of the infarct area, with loss of this no reflow effect, and cavitary dilatation.

Despite attempts at creating similar sized infarcts between animals and treatment groups, the groups had significantly different baseline infarct sizes, on the day of surgery (see Fig. 2A). The control group had infarcts involving 23 ± 4% of the LV, the bone marrow monocyte group, 14 ± 3%, and the stromal group, 34 ± 5%. In all cases, the ligature was placed in a similar anatomic location, just distal to the first diagonal branch.

Because of these variances, we normalized these differences by determining the relative degree of infarct size reduction from baseline over the course of 12 weeks to assist in the analysis of treatment effects. To determine the validity of this approach and in order to determine whether or not initial infarct size, measured immediately following surgery, was related to changes in infarct over time, we conducted Pearson product-moment correlations between infarct size at surgery and relative change in infarct size at all times by groups separately. There was only one significant correlation for the Stromal group between initial infarct and relative changes in infarct at week 1. That correlation was likely due to random experimental error. With 30 correlations performed, the probability of a significant correlation due to chance alone is 1.5, indicating that one and a half correlations would be significant due to chance alone. Thus, our analysis shows that there were no associations between initial infarct size and relative changes in infarct size over time. In order to increase the range of infarct size, a supplemental analysis was conducted that included cases for all groups at once. There were no significant associations observed. Thus, the infarct size at surgery does not predict changes in relative infarct reduction over time. Any statements made about treatment are not confounded by the initial infarct size.

Therefore, using the relative change as the index parameter, all groups had almost exactly the same degree of relative scar reduction up to the 6 week point, (control -62.4 ± 4.3%, stromal -64 ± 8.3%, and BMMC's -61.5 ± 5.6%) beyond which the curves began to diverge (Figure 2B). At 12 weeks, the control animals had a 75 ± 5% reduction in scar, stromal cells 67 ± 3%, and bone marrow monocytes 88 ± 5%, Using the 6 week time point as the reference point, there was a statistically significant difference between the degree of further infarct shrinkage beyond 6 weeks in the BMMC group in comparison to both the controls (p = 0.046) and stromal animals (p = 0.032) (Fig 2C). Figure 5 shows in a representative CMR of a dog heart, the infarct size reduction from week 1 to week 12.

Although there was an improvement in regional motion by approximately two wall motion scores in all groups (of those segments with a baseline score of 4 or less), there was no difference in the degree of improvement between treatments at 12 weeks (Figure 3).

For left ventricular volume (mass), there were modest differences between groups with small declines over time with the stromal animals having the greatest decrease between 8–12 weeks (presumably related primarily to infarct shrinkage) (Figure 4A). However, there was no difference in the increase in enddiastolic volume between groups at any time (Figure 4B).

As we did not quantify retained cell numbers using SPECT in these experiments, it is difficult to make any statements regarding correlation of cell numbers and treatment effects. This will be an important component of future experiments.

### Identification of PKH26 labeled cells at injection sites

**Figure 6:**
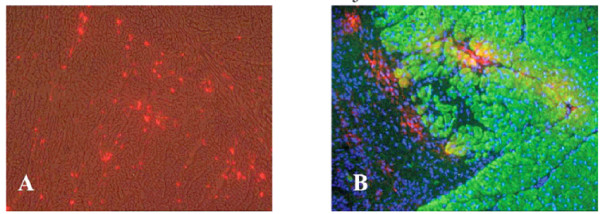
**Identification and characterization of PKH26+ (fluorescent) labeled donor cells in damaged myocardium 3 weeks following direct injection of Bone Marrow Mononuclear cells (BMMCs) into myocardium**. A) Overlay of PKH26 fluorescence (red) on 160× bright field image of unfixed cryostat section reveals bright red foci, which represent engrafted PKH26+ donor BMMCs. B) Overlay of beta cardiac myosin heavy chain immunofluoresence (IF = green) with PHK26 fluoresence (red) and Hoesch 33258 fluoresence (blue) reveals red donor cells at the interface between scar (left) and myocardium (right). Yellow MyHC+/PKH26 + BMMCs derived cardiomyocytes can also be observed. (Mag 160×).

In the three animals sacrificed at 3 weeks, most serial tissue sections showed PKH26 labeled cells interspersed with the India ink used to label injection sites. As shown in Fig. 6, Hoesch 33258 labeled nuclei and BMMC cells were observed within the scar tissue, as demonstrated by blue and red fluorescence, respectively. In some cases, red PKH26 labeled cells co-expressed with Mab4A9 labeled cardiac myosin heavy chain (MyHC) as indicated by yellow fluorescence in the overlay. This may be the result of BMMCs fusing with host cardiomyocytes and/or transdifferentiating into cardiomyocytes. PHK26 positive cells co-expressing MyHC were relatively rare, comprising approximately 2–4 cells per field. The presence of PKH26 positve cells would be in keeping with the Indium activity seen in the animals imaged with SPECT at 14 days and the clearance half-life of 5.3 days in the stromal experimental group.

When injection sites were examined from dogs euthanized 12 weeks post-injection, fluorescence microscopy detected PKH26 label associated with the extracellular matrix and individual cells. However, the relative number of labeled cells was much reduced from week 3 animals. Again, the small number of cells identified would be predicted based on the clearance kinetics observed with SPECT. It is difficult to comment on the correlation between cell numbers seen at 12 weeks and the treatment effect observed on CMR. In all cases, PKH26 label was observed near India Ink used to mark injection sites. In contradistinction to 3 weeks, immunofluorescent co-localization of MyHC failed to detect PKH26 positive cells which co-expressed MyHC.

### Effect of Cell Injection on Angiogenesis

**Figure 7:**
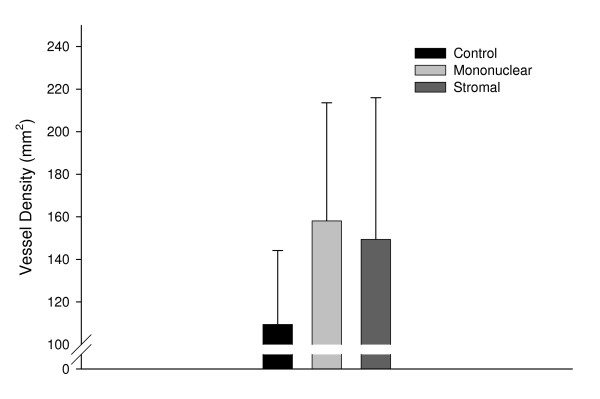
**Bar graph illustrating the vessel density (vessels/mm^2^) in the peri-infarct region of each experimental group**. A total of five random fields of view (400×) bordering the infarct scar had vessel structures counted for two injection sites for each animal in each group. The number of capillaries for each animal was averaged and calculated per square millimeters and used to calculate the vessel density for each group +/- SD (control n = 6, mononuclear n = 5, stromal n = 7). Of the three groups, the mononuclear and stromal stem cell treated animals showed approximately a 1/3 increase in the density of blood vessels within the peri-infarcted region compared to control animals that received an infarct but no cells, but was not significant due to the small number of animals per group (F 2,15 = 1.30; p = 30).

Of the three groups, the mononuclear and stromal stem cell treated animals showed approximately a 1/3 increase in the density of blood vessels within the peri-infarct region compared to control animals (Fig 7). This was not statistically significant (F (2,15) = 1.30; p = .30) perhaps due to the small sample size (15 animals per group would have been required if the same trends were maintained (Sample Power 2.0, SPSS inc. 2000)).

## Discussion

This study establishes the methodology for monitoring cell retention at the site of transplantation and determining the impact of these injections upon a) the natural change in infarct size, wall motion, and remodeling indices serially for a 12 week period. We have tracked cell retention using the radioactive tracer Indium^111 ^and the fluorescent lypophyllic marker, PKH26, to co-label our cells in vitro. Previously, we have shown that our labeling procedure, at the radioactive doses used, does not affect the survival, proliferation or differentiation of stromal cells [38]. There has been concern that Indium labeling may lead to harmful effects on cell function, but the administered dose per cell was not provided in that publication [35]. SPECT of In^111 ^has allowed us to evalaute cell clearance kinetics, up to 2 weeks, and to correlate these with measures of treatment effect. We observed a rapid loss of ^111^In signal over a two week period post-injection, and this correlated with a small number of PKH positive cells at 12 weeks. Since SPECT could not detect ^111^In signal at 12 weeks post-injection, a direct comparison between ^111^In signal and the number of PKH26 labelled cells was not possible. However, rapid cell loss has previously been described for muscle satellite cells injected into skeletal muscle [63] and for satellite cells injected into myocardium [64]. We do not know if this observed rapid clearance is the result of cell death caused by the relatively hostile inflammatory environment present in recently infarcted myocardium used in our model, the migration of cells away from the injection site, or if the kinetics described only apply to the cell lines used. We did not, in these experiments, quantify retained cell numbers and therefore, we are not able to correlate these with treatment effect.

While there was some evidence that BMMCs can transdifferentiate into cardiomyocytes at 3 weeks post-injection (Figure 6), this was a relatively rare event. We did not observe BMMC or stromal cell-derived cardiomyocytes in any of our treated dogs at 12 weeks.

Our study demonstrates a therapeutic effect from the injection of autologous BMMC's into the peri-infarct region after 3 hrs of ischemia and 3 hrs of reperfusion, when compared to controls or stromal cell-treated animals. Previous studies have demonstrated improved neovascularization following transplantation of monocytes in a murine model [65], a reduction in infarct size with increased angiogenesis but no change in regional function in a porcine model [66], alteration of LV remodeling indices in a rat model [67], and even improved angiogenesis and cardiac function when cells were retroperfused through the cardiac veins of pigs subject to a left anterior descending occlusion [68].

Stromal cells have been claimed to produce superior myocardial regeneration in rats [69,70], and a porcine model [71], but only increased angiogenesis with no improvement in scar reduction in a rat model [70]. These inconsistent literature reports leave uncertainty as to whether cell therapy does provide reproducible evidence of benefit in large animal models. It is our hope that the concurrent use of MR to monitor changes in scar shrinkage, and correlation of this with both cell retention kinetics and quantitative measure of cell retention (in future experiments), will help to provide more concrete evidence to support claims of treatment efficacy. Infarct shrinkage was the parameter, which, in our hands, was associated with the most consistent pattern of evolution, and the least inter-animal variation through to 6 weeks. Beyond that point, the curves began to diverge with a treatment effect with the BMMC's. We would suggest that future studies focus on this index parameter as a gauge of treatment response. Further, our study provides a framework for planning imaging studies to monitor the effect of cell therapy. We recommend early post-infarct imaging, a repeat at 6 weeks, and then at the end-point of treatment, which may be 12 weeks or longer. The pattern and degree of initial infarct shrinkage may also allow calculation of the necessary sample size needed to establish treatment effect.

Although Orlic claimed a major reduction in the extent of infarct size [6,8], others have not been able to reproduce these results using similar experimental methods [17,18]. While the cellular basis for improved cardiac function is still unknown, recent studies suggest that any therapeutic value may involve mechanisms that prevent ventricular dilation, increase myocardial wall thickness (resulting in improved cardiac output) and promote neo-angiogenesis at the site of injury.

We did not see any evidence in our study of cellular differentiation nor of fusion with existing constituents at 12 weeks. Rather, we witnessed the relatively rapid clearance of cells from the injection sites with a half-life of about 5 days, suggesting that these marrow-derived cells could only produce benefit through a transient paracrine effect that persisted beyond their clearance. The rapid loss of cells from injection sites has previously been described for other target tissues, such as skeletal muscle [70], and cell types such as cardioblasts [71], and is unlikely to be a consequence of radiation effects. However, Tran claimed no loss of cells from either infarct or normal myocardium from 2 hrs to 7 days after injection in a rat infarct model [72].

Also, Tran et al recently assessed in a rat model the usefulness of dual-isotope Tc-MIBI perfusion and Indium-oxine cell labeling imaging to gauge the pattern of infarct evolution [37]. They found no significant shrinkage in the size of the perfusion defect between the day of infarction and 1 month, in marked contradistinction to our CMR findings. We used CMR because of its superb spatial resolution [73-77] and it would appear to be a more reliable way of assessing infarct evolution than nuclear medicine-based measures of myocardial perfusion. These depend on sequestration of the perfusion tracer by metabolically intact cells. Our CMR findings in the control animals are consistent with those of Fieno et al who found that between 3 days to 4–8 weeks following infarction, there was a reduction in the extent of CMR signal enhancement to 24 +/- 3% of the original values [78], or in effect, a 76% reduction in scar size. Our study demonstrated a progressive reduction in infarct size of 75% in the control group at 12 weeks. Studies that look at a single time point, at sacrifice for example, may not appreciate the evolutionary changes that have occurred, and may potentially detect no differences in infarct size reduction with treatment if they have vastly different baseline.

## Conclusion

The present study showed minimal effects of cell therapy on left ventricular remodelling indices, with no substantive changes in regional function (using a relatively crude measure) with only a trend towards increased capillary density. However, this study does indicate that the combined use of non-invasive imaging modalities will allow the accurate assessment of this treatment through correlation of the evidence of engraftment, and retention, with indices of infarct size reduction and remodelling. Future studies, administering cells at different time points after infarction, should also include a means of quantitatively establishing the number of engrafted cells by calibrating the Indium images, and the concurrent assessment of the extent of perfusion abnormality and scar using CMR.

## Competing interests

The authors declare that they have no competing interests.

## Authors' contributions

GW and FP were involved in the conception and design, data analysis and interpretation, manuscript writing and final approval of manuscript. KL was involved in the collection and/or assembly of data, data analysis and interpretation and manuscript writing. PZ was involved in the collection and/or assembly of data. HK, RM, PZ, SD, CC, GW and JS were involved in the collection and/or assembly of data, data analysis and interpretation. PM was involved in the conception and design, data analysis and interpretation and manuscript writing. YB was involved in data analysis and interpretation.
